# Anisotropy Evaluation and Defect Detection on Laser Power Bed Fusion 316L Stainless Steel

**DOI:** 10.3390/mi14061206

**Published:** 2023-06-07

**Authors:** Zhixin Peng, Wei Xu, Yang Liu, Kai Zhao, Ping Hu

**Affiliations:** 1 School of Power and Mechanical Engineering, Wuhan University, Wuhan 430072, China; zhixin.peng@whu.edu.cn; 2 Shanghai Aerospace Equipments Manufacturer Co., Ltd., Shanghai 200245, China; 15821818200@139.com; 3 Institute of Technological Sciences, Wuhan University, Wuhan 430072, China; liuyang@whu.edu.cn

**Keywords:** metal additive manufacturing, anisotropy evaluation, defect detection, laser ultrasonics

## Abstract

Because of rapid heating, cooling, and solidification during metal additive manufacturing (AM), the resulting products exhibit strong anisotropy and are at risk of quality problems from metallurgical defects. The defects and anisotropy affect the fatigue resistance and material properties, including mechanical, electrical, and magnetic properties, which limit the applications of the additively manufactured components in the field of engineering. In this study, the anisotropy of laser power bed fusion 316L stainless steel components was first measured by conventional destructive approaches using metallographic methods, X-ray diffraction (XRD), and electron backscatter diffraction (EBSD). Then, anisotropy was also evaluated by ultrasonic nondestructive characterization using the wave speed, attenuation, and diffuse backscatter results. The results from the destructive and nondestructive methods were compared. The wave speed fluctuated in a small range, while the attenuation and diffuse backscatter results were varied depending on the build direction. Furthermore, a laser power bed fusion 316L stainless steel sample with a series of artificial defects along the build direction was investigated via laser ultrasonic testing, which is more commonly used for AM defect detection. The corresponding ultrasonic imaging was improved with the synthetic aperture focusing technique (SAFT), which was found to be in good agreement with the results from the digital radiograph (DR). The outcomes of this study provide additional information for anisotropy evaluation and defect detection for improving the quality of additively manufactured products.

## 1. Introduction

Additive manufacturing (AM) is a process of creating three-dimensional (3D) objects via layer by layer with the aid of design CAD software [1]. Compared with subtractive manufacturing, which is a traditional material removal technique using turning, milling, drilling, and other machining, additive manufacturing is more efficient and results in less material wastage. It is widely used in the fields of aerospace, automobile, electric power, and medical treatment, and additive manufacturing technology can be applied to most materials, such as metals, ceramics, polymers, composites, and sand. Among them, additive manufacturing of polymers and composites can be achieved by the fused deposition modeling (FDM) method [2], while the metal additive manufacturing techniques for metals are mainly based on directed energy deposition (DED) and powder bed fusion (PBF). According to the high energy beam used to melt metals, PBF can be divided into electron beam melting (EBM), selective laser sintering (SLS), the laser powder bed fusion (LPBF). SLS requires pre-melting of the binder in the raw material to complete the metallurgical bond, and LPBF is based on SLS. LPBF is also known as selective laser melting (SLM), and both are used to achieve additive manufacturing by selectively and completely melting the metal powder on the powder bed with the laser beam [3]. In recent years, metal additive manufacturing has been rapidly developed by virtue of its unique performance characteristics [4]. However, because of the rapid heating, cooling, and solidification during metal additive manufacturing (AM), the products exhibit strong anisotropy and are at risk of quality problems as a result of metallurgical defects [5,6]. The defects and anisotropy affect the fatigue resistance and the material properties, including mechanical, electrical, and magnetic properties, which limit further applications of the metal additively manufactured components in the field of engineering. Thus, it is important to evaluate the anisotropy and to perform defect detections in order to guarantee the quality.

So far, there has been plenty of research on anisotropy evaluation for materials, and the methods can be divided into destructive testing and nondestructive testing (NDT). Typically, destructive testing includes metallography, electron microscopy, X-ray diffraction (XRD), and electron back-scattering diffraction (EBSD), which could be used for obtaining accurate microstructural information for the samples. The microstructure of a selective laser melting Inconel 718 alloy in different directions was observed through the metallography, and the subcrystalline microstructure of laser additively manufactured 316L stainless steel was obtained through electron microscopy [7,8]. The alteration of the phase composition on an additively manufactured Ti6Al4V alloy after different surface finishing processes was analyzed using XRD, and the columnar grain orientation of the laser powder bed fusion CoCrMo alloys was characterized via EBSD [9,10]. These four destructive methods are often used in combination to obtain detailed microstructural information. However, the materials are often damaged and the experimental data regarding the components are often achieved from local analysis rather than global analysis. As a result, nondestructive testing is attracting increased interest because it could evaluate the material properties and detect existing defects without altering the integrity or the performance of a part. In the early stage of nondestructive testing, destructive testing is necessary to confirm the accuracy of the NDT results. For quantitative nondestructive evaluation, ultrasonic NDT is usually applied to evaluate the material anisotropy and to inspect defect detection with an absence of radiation, high detection efficiency, and sensitivity to volumetric flaws. Currently, ultrasonic nondestructive evaluation is based on analyzing the characteristics of ultrasound from the inspected object, including wave speed, ultrasonic attenuation, and diffuse backscatter results. For example, the longitudinal wave velocity was used to estimate the yield strength of the duplex stainless steel with different ferrite fractions as well as the mean grain size of metal matrix composites at different sintering temperatures [11,12]. Papadakis stated that the attenuation in polycrystalline materials could be classified as Rayleigh scattering, random scattering, and diffuse scattering according to the frequency and average grain size [13]. Then, he applied ultrasonic attenuation to characterize the distribution of the grain size and cavities for polycrystalline metals [14]. Subsequently, ultrasonic attenuation was employed for assessment of the average grain size, the inhomogeneous grain growth process, and the grain size distribution for various metals [15,16,17]. On the other hand, the received backscattered signals were utilized for characterizing the microstructure. Earlier backscattering models were focused on simple polycrystalline media with cubic, equiaxed, and single-phase grains [18,19]. With more realistic complex polycrystals with elongated, duplex, or multiple phase grains, such as from the casting, welding, and additive manufacturing processes, the corresponding diffuse backscatter models were proposed for the ultrasonic characterization [20,21,22,23,24]. Specifically, the diffuse backscatter results successfully qualified the grain size distribution and the degree of anisotropy of railroad wheels [25,26]. Zhang et al. estimated the grain size with ultrasonic backscatter, longitudinal, and transverse wave attenuation via immersion transducers, ultrasonic arrays, and PZT plates, respectively [27]. Choi et al. compared the linear ultrasonic methods with the nonlinear ultrasound for correlating the grain size and mechanical properties of the materials [28]. In the field of metal additive manufacturing, similar applications have been performed. Sol et al. investigated the anisotropy of the additively manufactured components, in which it was found that transverse velocity and ultrasonic attenuation were sensitive to the build directions [29]. Kim et al. used ultrasonic phase velocity to assess the anisotropy of additively manufactured 316L [30]. Sotelo et al. evaluated the additively manufactured components using the wave speed, attenuation, and ultrasonic backscattered results from the traditional ultrasonic testing, which agreed with the microhardness and destructive measurements [31]. Among them, the materials that were mostly focused on were from traditional material processing rather than additive manufacturing. In addition, for the microstructures of the metal additive manufactured components, the anisotropy was shown in different directions. Here, three methods, namely acoustic velocity, attenuation coefficient, and ultrasonic backscattering, are under investigation.

For guaranteeing the quality of metal additive manufacturing, flaw inspection is necessary. However, anisotropy affects the identification of the defect. For reducing the effect of microstructure anisotropy on the defect detection of additively manufactured components, Li et al. subsequently proposed a total focus method using an ultrasonic annular array to improve the detection on the TC 18 titanium alloy additively manufactured components and accurately detected artificial defects with a diameter of 0.8 mm [32]. Because of the complex production environment, the ultrasonic phased array method is difficult to adapt for in situ online monitoring for metal additive manufacturing. Thus, the non-contact and long-range laser ultrasonic method shows promising application in this field [33]. The synthetic aperture focusing technique (SAFT) on ultrasonic imaging can be used to reduce the interference of the microstructure of additively manufactured components. Lévesque et al. used the laser ultrasonic method with SAFT to detect the lack of fusion and porous defects for In 718 and Ti-6Al-4V additively manufactured components, and then applied a similar method to detect delamination defects for cold sprayed additively manufactured components [34,35]. The defect location could be recognized, while the specific shape of the defects was not easy to identify. From this point of view, the artificial defects helped to improve the defect detection. Lv et al. applied a 3D SAFT with laser ultrasonic Rayleigh waves to focus on the damage area and the subsurface artificial defect detection of both AlSi10Mg and 316L additively manufactured components, in which the inspection images of the subsurface defects were clearly identifiable [36]. In this article, different types of artificial defects with smaller sizes are detected via laser ultrasonics.

The microstructure properties and macroscopic defects of materials are two important factors related to the quality of metal additive manufacturing. In this article, the microstructure information of the laser power bed fusion 316L stainless steel is first measured using conventional destructive approaches with metallographic methods, X-ray diffraction (XRD), and electron backscatter diffraction (EBSD). Then, the anisotropy of the laser power bed fusion 316L stainless steel is evaluated through ultrasonic nondestructive characterization using the wave speed, attenuation, and diffuse backscatter results. In addition, a synthetic aperture focusing method of laser ultrasonics will be utilized to identify the artificial defects, which are produced by printing the laser power bed fusion 316L stainless steel. Here, artificial defects with different sizes and shapes were created. Finally, the applicability of the laser ultrasonic technique based on SAFT for detecting the defects of metal additively manufactured components was verified using a digital radiograph (DR). This article provides a reference for further improving the component quality by considering both anisotropy evaluation and defect detection for metal additively manufactured components.

## 2. Materials and Methods

### 2.1. Sample Preparation

**Figure 1:**
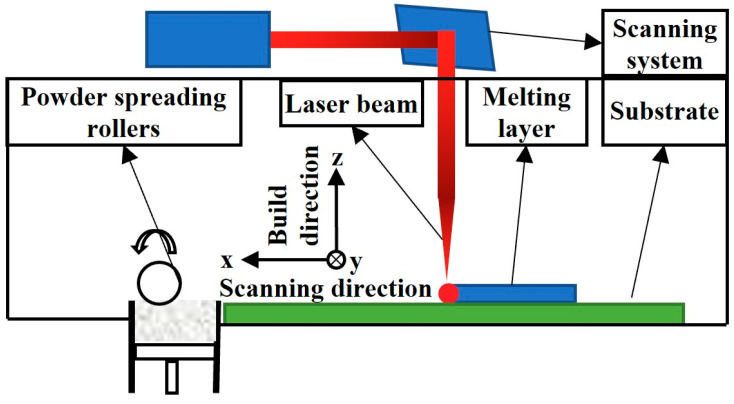
Schematic diagram of the LPBF process.

The 316L stainless steel sample was prepared using laser power bed fusion (LPBF) additive manufacturing, as shown in Figure 1. The build direction was along the additive manufacturing, which was set as the *z* direction, and the scanning directions vertical to the build direction, which were set as *x* and *y*, respectively. The metal powder of the raw material of the additive manufacturing had a diameter of 15 μm–52 μm. During the LPBF process, the laser power was 300 W, the scanning speed was 650 mm/s, and the thickness of a single layer was 50 μm, in which each layer was rotated 67° before scanning the next layer, and the scanning distance between adjacent the passes was 140 μm.

**Figure 2:**
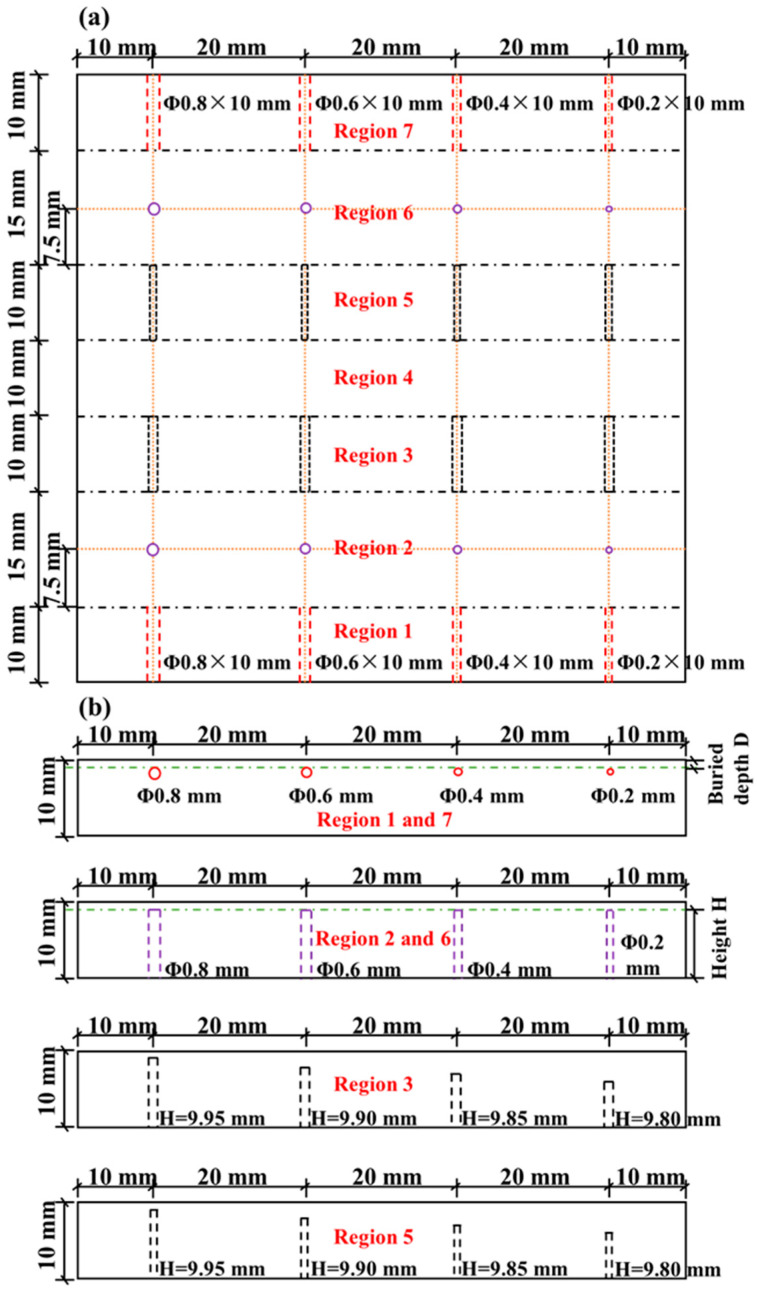
Artificial defect design of the additively manufactured block: (**a**) top view and (**b**) main view.

Two different sizes of laser power bed fusion 316L stainless steel were prepared. One was a cube sized 24 mm × 24 mm × 24 mm and the other was a block sized 80 mm × 80 mm × 10 mm. The cube was inspected for ultrasonic nondestructive characterization using the wave speed, attenuation, and diffuse backscatter results through ultrasonic testing, while the block was used to study the effect of the microstructure on defect detection using laser ultrasonics. The additively manufactured components without any treatment had a rough surface that contained great noise interference and reduced the signal-to-noise ratio. Several different levels of grit sandpapers were used to polish the surface of the samples for excluding the interference of surface roughness. The cube was also polished for removing the influence of the surface roughness. For simulating the real situation as much as possible, the artificial defects were produced during additive manufacturing, which were created at various locations, depths, and diameters of side-drilled holes, flat-bottomed holes, and notches, as shown in Figure 2. The additive manufacturing process was controlled by importing the corresponding 3D model to the operating software, which directly printed the artificial defects designed by the drawing. Because of the complex temperature variations and melt pool behavior of the additive manufacturing, accurate control of size tolerances of the artificial defects is currently very difficult and subsequent inspections are required. To better illustrate the experimental results, the top view of the block was assumed to be divided into seven regions according to the type and location of the artificial defects. Regions 1 and 7 had two sets of side-drilled holes with buried depths of 0.2 mm and 0.1 mm, respectively, both with lengths of 10 mm. The diameters of each set of side-drilled holes were 0.8 mm, 0.6 mm, 0.4 mm, and 0.2 mm from left to right, respectively. Regions 2 and 6 had two sets of flat-bottomed holes with heights of 9.8 mm and 9.9 mm, respectively. The diameters of each set of flat-bottomed holes were 0.8 mm, 0.6 mm, 0.4 mm, and 0.2 mm from left to right, respectively. Regions 3 and 5 had two sets of notches with widths of 0.5 mm and 0.2 mm, respectively, and both had lengths of 10 mm. The heights of each set of holes were 9.95 mm, 9.90 mm, 9.85 mm, and 9.80 mm from left to right, respectively. Region 4, without any artificial defects, remained and was used for comparison with other defective regions. The artificial side-drilled holes, flat-bottomed holes, and notches were used to exam the laser ultrasonic detection on different forms of metallurgical defects, such as cracks, holes, and unfused defects occurring in the metal additive manufacturing process.

### 2.2. Ultrasonic Nondestructive Characterization

The ultrasonic nondestructive characterization was operated for the wave speed, attenuation, and diffuse backscatter results through ultrasonic measurement of a UPK-T36 system (MISTRAS, Princeton Junction, NJ, USA), in which the transducer (OLYMPUS, 5 MHz, 50.8 mm point target focus, and 12.7 mm element diameter) was connected to a DPR 300 pulser/receiver (JSR Ultrasonics, Pittsford, NY, USA).

The wave speed is one of the most basic ultrasonic parameters. For polycrystalline materials, the wave speed exhibits anisotropy at the macroscopic level when there is a significant crystal growth orientation within the building process. In this article, the three directions of the additively manufactured component were tested using the longitudinal waves. The time interval τ between the primary and secondary bottom echoes was the time taken for the ultrasound to pass through twice the thickness of the sample. The method was used to obtain the corresponding correlation function of the primary and secondary bottom echoes signals, find the sampling point corresponding to the peak position of the correlation function, and calculate the time interval τ using the relationship between the sampling point and the sampling frequency.

When ultrasonic waves were propagated in the media, a reduction in the sound pressure and sound energy occurred, which is called the propagation of ultrasonic attenuation. Two mechanisms of ultrasonic attenuation were scattered from the microstructure/flaws and absorption due to dissipation. For metal materials, the scattering attenuation mainly dominates, and the absorption attenuation can usually be ignored. The attenuation coefficient is not a constant value, but a function of frequency. Here, the fast Fourier transform (FFT) method was used for calculating the frequency-dependent attenuation coefficient α(f).

When an ultrasonic wave propagates inside the media, it encounters the interface with different acoustic impedance, such as the grain boundaries and the phase interfaces, then ultrasonic scattering occurs. Within heterogeneous media, the observed grain noise in pulse–echo-type experiments is often called “backscatter”, in which the received scattering is in the opposite direction of the incident wave propagation. The ultrasonic backscatter signal is located between the frontwall and backwall echoes. As the orientation and size of the grain are various at different locations in one sample, the backscattered signals are correspondingly different. In addition, ultrasonic backscatter signals are related to the grain size, impurities, porosity, and so on. Thus, multiple testing areas are selected on the surface of the specimen during ultrasonic backscatter measurements.

### 2.3. Laser Ultrasonics for Defect Detection

**Figure 3:**
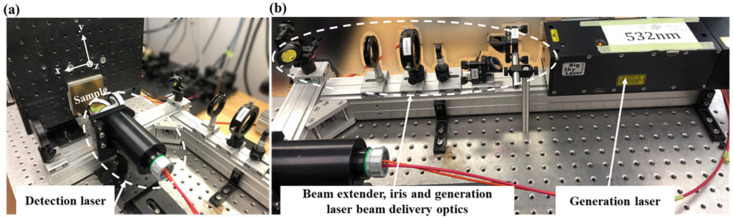
Laser ultrasonic experimental setup: (**a**) generation laser and optical path unit, and (**b**) detection laser and sample.

Defect detection was performed using a Lus Advance laser ultrasonic inspection system (Tecnar, Hocquart Street Saint-Bruno, QC, Canada), as shown in Figure 3. The CFR 200 generation laser (Lumibird, Lannion, France) had a laser wavelength of 532 nm and the LUS-05 inspection laser had a laser wavelength of 1064 nm. The generating laser excited the ultrasonic waves through the thermoelastic effect [37], while the detection laser was connected to a two-wave beam mixing interferometer. The ultrasonic signals were received by means of optical interference, which improved the signal-to-noise ratio for detection on relatively rough surfaces. During the experiments, the laser ultrasonic device was scanned in the *x*–*y* plane.

Because of the complex thermal cycling process, the microstructure of metal additively manufactured components has significant anisotropy and inhomogeneity that affect the defect detection results via laser ultrasonics. Here, the synthetic aperture focusing method was used to improve the inspection results. The principle of the synthetic aperture focusing method is that the received defect signals at each scanning position were superimposed according to the time delay, which could be equated to a large size ultrasonic transducer, thus essentially improving the lateral resolution of defect imaging [38].

**Figure 4:**
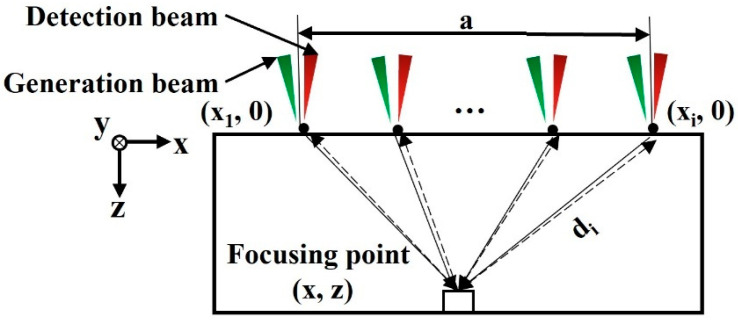
Schematic diagram of the synthetic aperture focusing method.

The specific implementation of the synthetic aperture focusing method is shown in Figure 4. The ultrasound is excited and received at the surface opposite to the defect. The laser sweeps in the *x*–*y* plane with a certain scanning step. Each sweeping point is noted as (*x_i_*, 0), where *i* = 1, 2, …, *n* (*n* is the total number of scanning points in the *x* direction), and the synthetic aperture focusing point is noted as (*x*, *z*). The ultrasound at each sweeping point reaches the focus and is reflected back in the original path. Then, the ultrasound propagation time during this period is denoted as *t_i_*, and *t_i_* can be calculated as shown in Equation (1),

(1) $$t_{i\;}=\frac{2\sqrt{z^{2}+{\left(x-x_{i}\right)}^{2}}}{v}=\frac{2d_{i}}{v}$$

where *d_i_* is the distance from the scanning point to the focus point, and *v* is the ultrasonic velocity. Then, the intensity *I*(*x*, *z*) of the focus point can be superimposed by signal *S_i_*(*t_i_*) of each scanning surface point at moment *t_i_*, and the calculation formula is shown in Equation (2),

(2) $$I\left(x,z\right)=\sum_{i=1}^{n}S_{i}\left(t_{i}\right)$$

The focus point is usually referred to as the location of the defect, and the intensity of the focus point is determined by the signal of each scan point at $t_{i}$, which is affected by the location of the scan point and the defect, as well as the wave speed, thus enabling focused imaging of the defect. The lateral resolution Δx and the vertical resolution Δz of the synthetic aperture focusing method can be calculated using Equations (3) and (4),

(3) $$\Delta x\;\approx \;v\Delta t\frac{z}{a},$$

(4) $$\Delta z\approx \frac{v\Delta t}{2},$$

where Δt is the ultrasonic pulse duration and *a* is the size of the synthetic aperture.

Finally, the digital radiograph (DR) inspection technique was selected to verify the defect detection results of the laser ultrasonics. The DR inspection system mainly included a ray source, flat panel detector, and control system. In order to obtain high signal-to-noise ratio images, the tube voltage and current of the ray source were 78 kV and 5 mA, respectively, and the pixel size of the flat panel detector was 200 μm. Additionally, the focal length, focus size, and magnification were set to 1100 mm, 0.4 mm, and 1.1 times, respectively.

## 3. Results and Discussion

### 3.1. Microstructure by Destructive Testing

In this section, the anisotropy of the laser power bed fusion 316L stainless steel components was first measured using conventional destructive approaches with metallographic methods, X-ray diffraction (XRD), and electron backscatter diffraction (EBSD). The metallographic method could be directly used to observe the microstructure morphology, in which the samples were under the operation of rough grinding, fine grinding, polishing, and etching in turn. The samples were first put on the polishing machine and were then taken from the chemical etching of the ferric chloride hydrochloric acid reagent and electrolytic etching of the 10% oxalic acid solution, respectively. Finally, the samples were placed under the optical microscope for observation.

**Figure 5:**
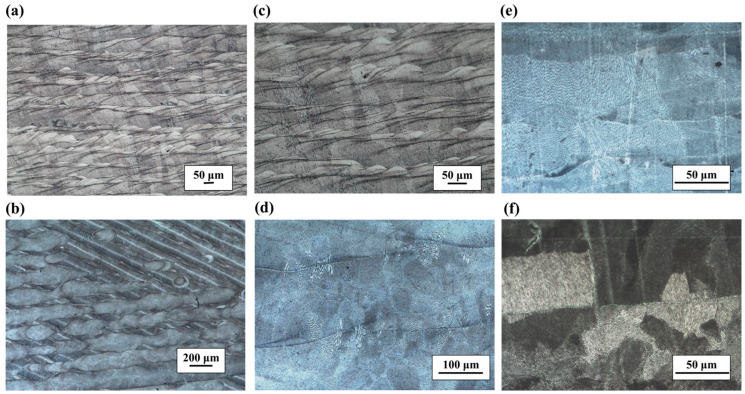
Microstructure of different orientation planes: (**a**,**c**,**e**) for *y*–*z* plane and (**b**,**d**,**f**) for *x*–*y* plane.

The metallographic microstructures in the *y*–*z* plane (vertical *x* direction) and *x*–*y* plane (vertical *z* direction) are shown in Figure 5, where Figure 5a,c,e corresponds to the microstructure in the *y*–*z* plane and Figure 5b,d,f is related to the microstructure in the *x*–*y* plane. Regarding the *y*–*z* plane, the metallography of Figure 5a,c is operated using chemical etching. Layers of fish-scale stacking can be easily seen, while the fusion lines of the adjacent layers are interlaced together, which makes it difficult to distinguish the interlayer interface. Because of the influence of powder size, scanning speed, laser power, and other parameters, the fusion line of the interlayer is not exactly parallel, and the interlayer thickness is obviously larger than the single thickness of the metal laying powder. Each layer of the passes consists of two parts, light and dark, in which the dark passes are angled approximately 20° towards the direction of the build. It is shown all the passes except the top layer are remelted with the latter one during additive manufacturing scanning. In Figure 5e, the different orientations of the dark and bright bands can be more clearly observed through electrolytic etching. On the *x*–*y* plane, the metallography in Figure 5b,d is finished with chemical etching. The microstructure consists of “weld paths”, in which the width of the weld paths and the cross angle of the weld paths of different layers are similar to the scan spacing and rotation angle of the process parameters, respectively. After electrolytic etching, as shown in Figure 5f, it is found that some grains are truncated by the weld path boundaries, but some continue to grow, which might be due to the intersection of adjacent weld paths during the melting–solidification process, as well as secondary remelting.

**Figure 6:**
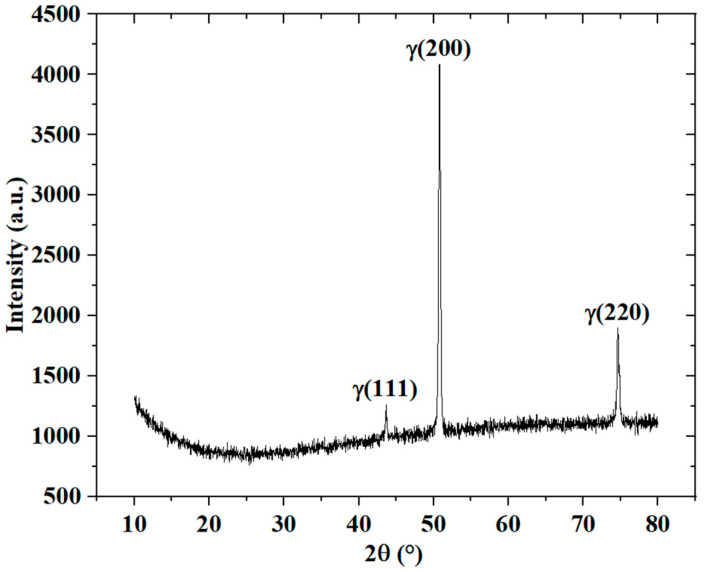
The X-ray diffraction analysis of the sample.

**Figure 7:**
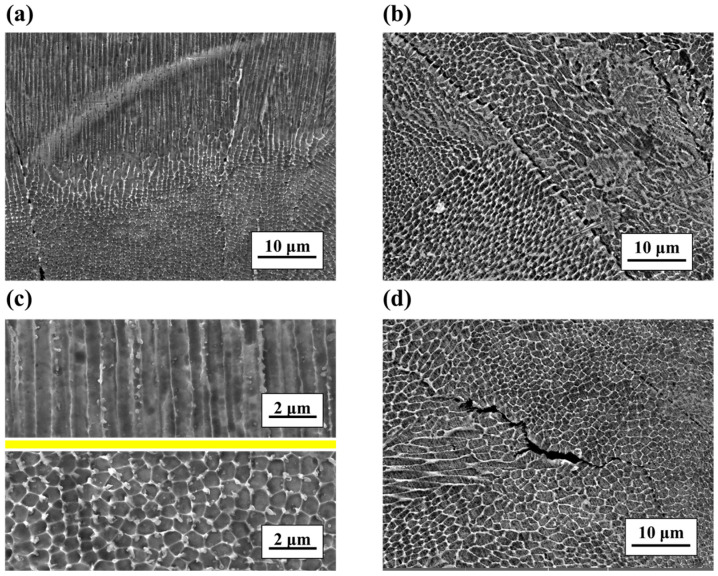
The scanning electron micrographs of different planes: (**a**,**c**) for *y*–*z* plane and (**b**,**d**) for *x*–*y* plane.

The physical phase composition of samples was analyzed by X-ray diffraction, as shown in Figure 6. The phase composition of the laser power bed fusion 316L stainless steel components is mainly γ-austenite single-phase organization, and the diffraction “triple peaks” of the (111), (200) and (220) crystal planes are marked in Figure 6. For further details, scanning electron microscopy (SEM) was applied to observe the microstructure. The electron microscopy scans in the *y*–*z* plane are shown in Figure 7a,c, while the electron microscopy scans in the *x*–*y* plane are shown in Figure 7b,d. In the *y*–*z* plane, the dark band and the bright band in the previous metallographic phase showed that the former was columnar and the latter was regular-cell-like, as shown in Figure 7a. The specific morphology of the columnar and cell-like results is shown in Figure 7c. In the *x*–*y* plane, the dark and bright areas in the previous metallographic phase showed that the dark area within the weld paths was in the form of a cell-like shape that approximated an equiaxed crystal, while the bright area at the boundary of the weld paths was in the form of an elongated strip, as shown in Figure 7b,d. According to the grain growth theory, this might be due to the temperature gradient of the weld path during additive manufacturing. The temperature inside the weld paths was evenly distributed, then the grains grew in all directions, while the temperature distribution at the weld path boundary was non-uniform, and the grains then grew in the opposite direction of the heat flow.

**Figure 8:**
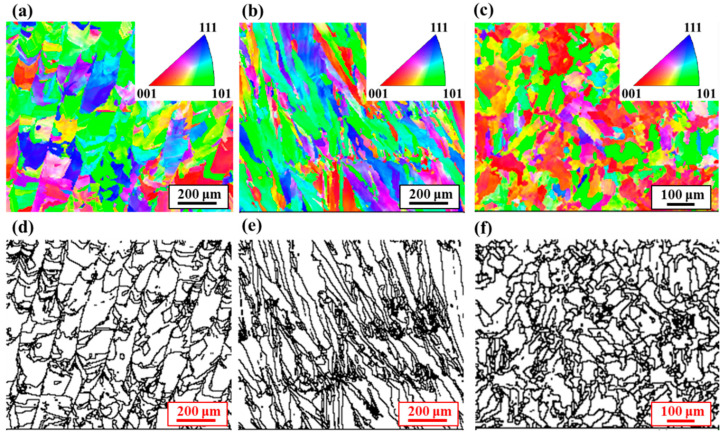
Inverse pole figures and grain boundary maps for EBSD analysis in different directions: (**a**–**c**) inverse pole figures, (**d**–**f**) grain boundary maps, (**a**,**d**) *x* direction, (**b**,**e**) *y* direction, and (**c**,**f**) *z* direction.

The orientations of grains in the *x*, *y*, and *z* directions were analyzed using the electron backscatter diffraction (EBSD) method, in which an orientation angle difference of 10° was selected for processing the diffraction results. Meanwhile, the grains with less than 10-pixel points were removed to reduce the pixel error. The inverse pole figure and grain boundary diagram in the *x* direction (corresponding to the *y*–*z* plane) are shown in Figure 8a,d. The grain orientation is relatively uniformly distributed with an average grain diameter of 22.52 μm. The columnar crystals are at an oblique angle to the build direction. The inverse pole figure and grain boundary diagram in the y direction (corresponding to the *x*–*z* plane) are shown in Figure 8b,e. The grain orientation is also relatively uniform with an average grain diameter of 20.99 μm. The inverse pole figure and grain boundary diagram in the *z* direction (corresponding to the *x*–*y* plane) are shown in Figure 8c,f. Compared with the *x* and *y* directions, the microstructure in the *z* direction is mainly in the (001) and (101) crystal planes, in which the average diameter of the grain is around 24.00 μm. The average grain diameters obtained using EBSD analysis in different directions are shown in Table 1, and the average grain diameters were obtained by fitting the log-normal distribution model to the calculation.

From the above the experimental results, it is shown that there are obvious columnar crystal organizations in the *y*–*z* and *x*–*z* planes, and the columnar crystals are at a certain oblique angle to the build direction. The microstructure in the *x*–*y* plane is the closely arranged weld paths, and the internal part of the weld paths is approximately composed of equiaxed grains.

### 3.2. Ultrasonic Characterization

Five positions, A, B, C, D, and E, were evenly selected on the corresponding planes of each direction of the additively manufactured component, and the wave speeds were measured five times in each direction. The wave speeds in the three directions calculated via the cross-correlation method are listed in Table 2, respectively. The coefficient of variation is utilized to describe the fluctuation of data, which is the ratio of the standard deviation to the mean. In the same direction, the wave speeds are varied at different measurement locations, in which the largest deviation is in the *x* direction with a coefficient of variation of 0.05%. The average value is used as the wave speed in the corresponding direction. Here, the effect of microstructure on the wave is not obvious. The sound velocity is mainly determined by the density, Poisson’s ratio, and elastic modulus of the material, which are not high enough to cause a significant change in the sound velocity.

**Figure 9:**
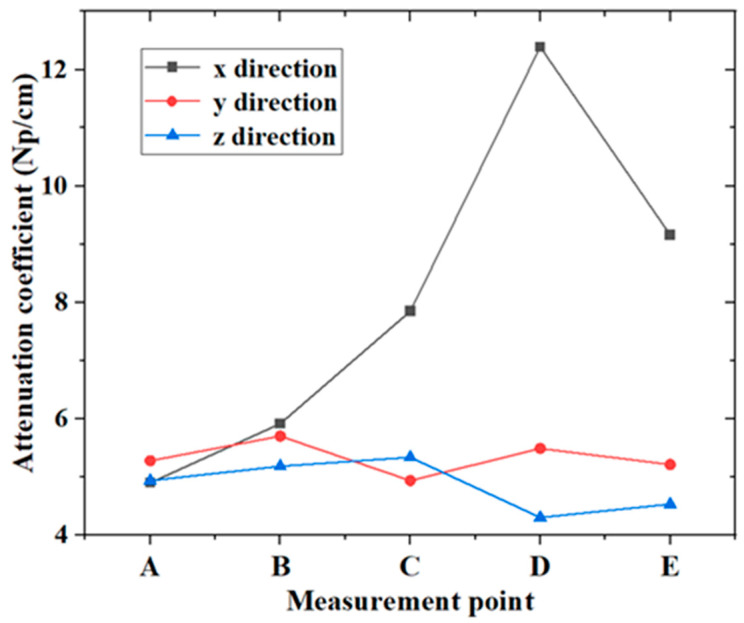
Measured values of attenuation in different directions.

Similarly, five positions, A, B, C, D, and E, were chosen for the measurement of attenuation on the corresponding planes for each direction. The attenuation in the three directions are shown and listed in Figure 9 and Table 3, respectively. In the same direction, the attenuation at different measuring locations are various, in which the largest deviation is in the *x* direction with a coefficient of variation of 32.84%. Compared with the wave speed, the attenuation is more sensitive to the microstructure. With the same central frequency of the transducers, the *z* direction has the largest mean grain diameter in the *x*–*y* plane, but has the smallest attenuation. From the results of EBSD, the shape of the grain in the *z* direction is approximately equiaxed crystal, and those in the *x* and *y* directions are mainly columnar crystals with a certain inclination angle to the build direction. When ultrasonic waves propagate in the *z* direction, the propagation direction is actually parallel to the build direction, which is along both sides of the columnar crystal. Then, the smallest attenuation in the *z* direction probably contributes to the relatively less interfaced with the grain boundary. On the other hand, when ultrasonic waves propagate in the *x* and *y* directions, the ultrasonic waves propagate perpendicular to the build direction, which is along the cross section of the columnar crystal. Thus, more scattering occurs because of the grain boundaries, which results in a large attenuation in the *x* and *y* directions, as measured.

**Figure 10:**
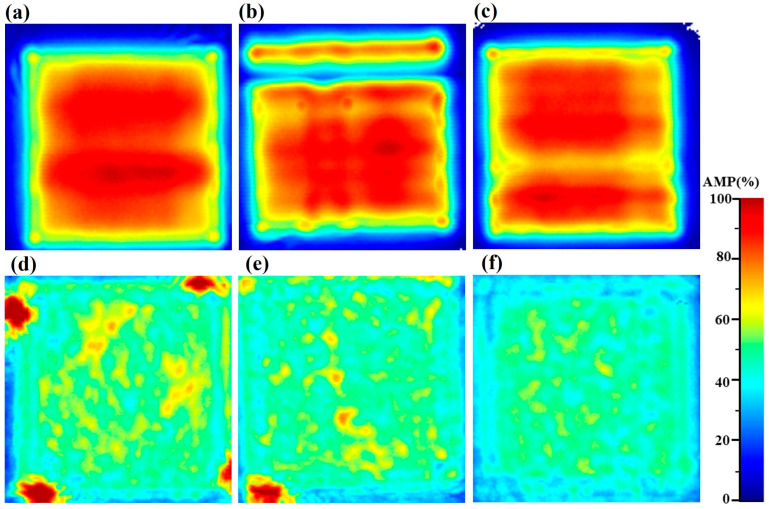
C-scan of backwall and diffuse backscattered in different directions: (**a**–**c**) backwall, (**d**–**f**) diffuse backscattered, (**a**,**d**) *x* direction, (**b**,**e**) *y* direction, and (**c**,**f**) *z* direction.

In order to investigate the effect of the microstructure on ultrasonic wave propagation, the diffuse backscatter and the backwall are shown in Figure 10, in which the results are obtained in the *x*, *y*, and *z* directions, respectively. In the C-scan of the backwall, the view is not uniform, especially in the y direction where there is a lower line region. This indicates that metallurgical defects exist at this location. In the diffuse backscatter results, the amplitudes in the *x* and *y* directions are both higher than the one in the *z* direction. The results agreed with the destructive results shown for EBSD, but with the global detection. Compared with the previous wave speed, it also shows that the anisotropy of the additively manufactured 316L stainless steel is more intense compared with the diffuse backscatter results.

### 3.3. Defect Detection

The laser ultrasonic approach was applied to inspect additively manufactured 316L stainless steel components with a series of artificial defects. Specifically, the effective width of the laser-generated ultrasonic probing pulse was measured to be 0.1 microseconds. The effective broadening was caused by scattering from the material, significantly reducing the ultrasonic bandwidth of the probing pulse from 50 MHz, as typically seen in the 316L sample, down to 10 MHz, as seen in the experiment. The pulse travel time in the bulk of the material was 3.5 microseconds for a sample thickness of 10 mm. Therefore, the effective velocity of the ultrasonic pulse in the material was calculated to be 5710 m/s. The lower velocity was due to the porosity within the material. Using this measured velocity, the depth resolution was estimated at 0.29 mm. The SAFT method was applied to the raw data to increase the lateral resolution of the ultrasonic image along the vertical axis and to increase the overall contrast of the image. The synthetic aperture size was selected to have an increased resolution of a factor of 5 for the backwall echo; that is, for *z* equal to the thickness of the sample. With an ultrasonic velocity of steel of 5710 m/s, the vertical resolution of the image was estimated at 0.114 mm. Note the SAFT method was applied along only one axis, namely the vertical axis of the image displayed.

**Figure 11:**
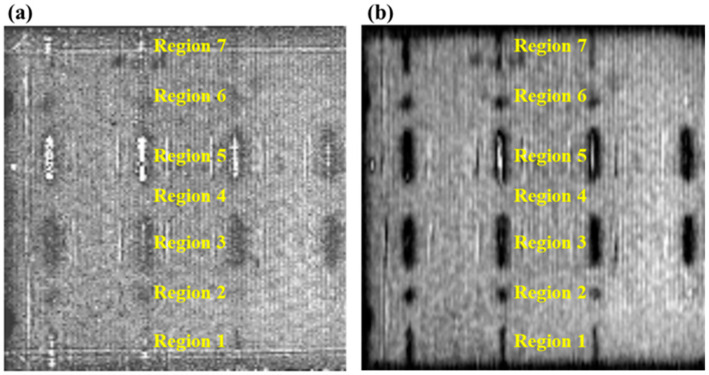
Laser ultrasonic C-scan results: (**a**) original image with raw data and (**b**) improved image with SAFT.

As shown in Figure 11a,b, the raw data and the improved image with the synthetic aperture focusing method are plotted, in which the regions are designed as shown in Figure 2. In Figure 11a, for regions 1 and 7, only the side-drilled holes with diameters of 0.8 mm, 0.6 mm, and 0.4 mm could be faintly visible, while the defect with a diameter of 0.2 mm cannot be identified. Similarly, the inspection results of regions 2 and 6 show blurredly flat-bottomed holes with diameters of 0.8 mm, 0.6 mm, and 0.4 mm, but the defect with the diameter of 0.2 mm cannot be recognized. These two holes with a diameter of 0.2 mm need to be double checked in the following digital radiograph (DR). The notches in both region 3 and region 5 can be totally confirmed. As shown in Figure 11b, the image is reconstructed via the synthetic aperture focusing method. Compared to the image with the raw data, most of the defects with flaws in regions 1, 2, 6, and 7 could be clearly observed, except for the diameter of 0.2 mm. It is worth mentioning that the diameters of 0.8 mm, 0.6 mm, and 0.4 mm could be more clearly visible. In regions 3 and 5, all the notches can be found, but the diameters of 0.5 mm and 0.2 mm are not quite different, which also need to be double checked in the following analysis of DR testing.

**Figure 12:**
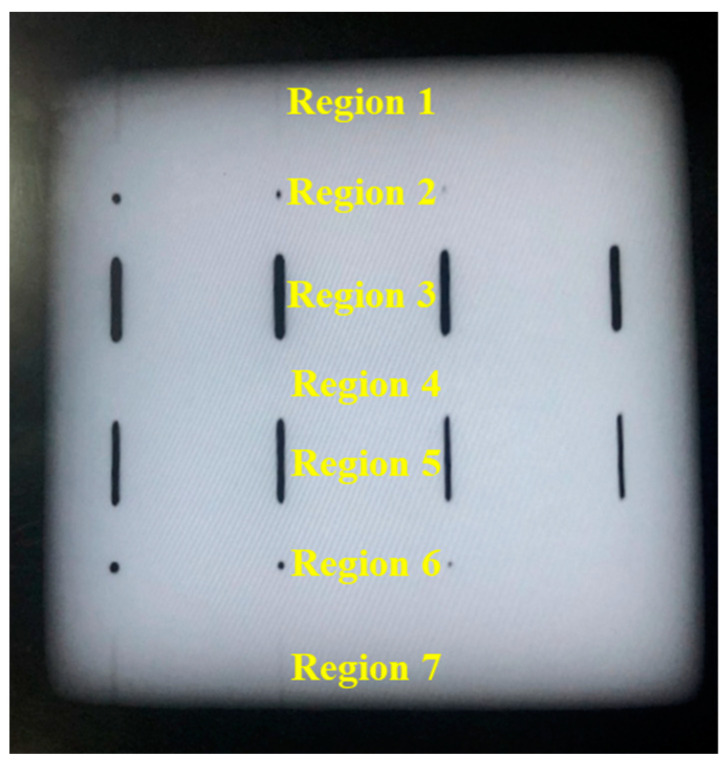
The result of the digital radiograph inspection.

To verify the results of the laser ultrasonics, digital radiograph (DR) was utilized and the inspection results are shown in Figure 12. It is found that the DR results agree well with the above laser ultrasonic results. For different regions, the defects are clearer for both locations and shapes when compared with the previous laser ultrasonic results, which is the advantage of DR technology. Specifically, the undetected flaws, which were originally designed, still have not been determined. In regions 3 and 5, the left two rows are almost exactly the same. This might be due to the forming quality during additive manufacturing. Thus, laser ultrasonic is a good choice for the additively manufactured components, especially with SAFT for decreasing the effect of anisotropy.

As stated above, defect detection experiments were used to evaluate the detection capability of laser ultrasound for additively manufactured components. The original image with raw data does not clearly show the location and shape of defects due to the effect of microstructure on the ultrasound signal. These conclusions have contributed to improving the application of laser ultrasonics for the quality inspection of additive manufacturing. With the proposed smart manufacturing and demand for in situ and online inspection of additive manufacturing, non-contact and long-range laser ultrasonic inspection has great potential in this field. The next plan is to explore the defect detection capability of laser ultrasound for different kinds of metal additive manufacturing components in order to expand the applicability of laser ultrasound.

## 4. Conclusions

In this article, the anisotropy of laser power bed fusion 316L stainless steel components was evaluated using a conventional destructive method with metallographic methods, X-ray diffraction (XRD), and electron backscatter diffraction (EBSD). The microstructure information of the additively manufactured components in three directions was obtained in the destructive testing results, in which the columnar crystal appeared through multiple layers in the *y*–*z* plane, and the grains were approximately equiaxed in the *x*–*y* plane. The destructive testing was compared to the ultrasonic nondestructive characterization using the wave speed, attenuation, and diffuse backscatter results through traditional ultrasonic measurement with an immersion transducer. The variation in the wave speed was not enough to verify the anisotropy of the additively manufactured components, while the attenuation and the diffuse backscattered results were more sensitive to the microstructure, in which the smallest attenuation was in the *z* direction, while the largest value was in the *x* direction. The C-scan images of the backscatter results showed that the amplitude of diffuse backscatter in the *x* and *y* directions was higher than the one in the *z* direction, which indicates higher anisotropy and is consistent with EBSD. Furthermore, to evaluate the detection capability of the laser ultrasonics, defects of side-drilled holes, flat-bottomed holes, and notches were pre-printed with various sizes during the additive manufacturing of the laser power bed fusion 316L stainless steel. Then, the raw data were analyzed using the synthetic aperture focusing method for improving the distinguishing by reducing the effect of the anisotropy. The laser ultrasonic results were verified through digital radiograph inspection. The outcomes of this study provide additional information for anisotropy evaluation and defect detection for improving the quality of additively manufactured products. In future work, the probability of detection and different kinds of microstructures for the additively manufactured components should be measured with the laser ultrasonic for expanding its applications. In addition, the anisotropy of the additively manufactured components should be tested using mechanical methods, and the results should be compared with the wave velocity, attenuation, and diffuse backscatter. Ultimately, a link between macroscopic mechanical properties and ultrasonic signals should be established in order to achieve a comprehensive quality assessment of the additively manufactured components.

## Figures and Tables

**Table 1:** Average grain diameter measured using EBSD.

Measurement Direction	Average Grain Diameter (μm)
*x*	22.52 ± 0.52
*y*	20.99 ± 0.35
*z*	24.00 ± 0.85

**Table 2:** Wave speed measurements in different directions.

Direction	*v_A_* (m/s)	*v_B_* (m/s)	*v_C_* (m/s)	*v_D_* (m/s)	*v_E_* (m/s)	Average Value (m/s)	Standard Deviation (m/s)	Coefficient of Variation (%)
*x*	5793.9	5799.4	5791.1	5793.9	5793.9	5794.44	2.71	0.05
*y*	5800.6	5806.1	5803.3	5803.3	5800.6	5802.78	2.05	0.04
*z*	5798.1	5800.8	5800.8	5800.8	5795.5	5799.20	2.13	0.04

**Table 3:** Attenuation of different directions.

Direction	*α_A_* (Np/cm)	*α_B_* (Np/cm)	*α_C_* (Np/cm)	*α_D_* (Np/cm)	*α_E_* (Np/cm)	Average Value (Np/cm)	Standard Deviation (Np/cm)	Coefficient of Variation (%)
*x*	4.9	5.9	7.8	12.4	9.2	8.04	2.64	32.84
*y*	5.3	5.7	4.9	5.5	5.2	5.32	0.27	5.10
*z*	4.9	5.2	5.3	4.3	4.5	4.84	0.39	8.01

## Data Availability

Data sharing is not applicable to this article.
